# Novel Time-Resolved Fluorescence Immunochromatography Paper-Based Sensor with Signal Amplification Strategy for Detection of Deoxynivalenol

**DOI:** 10.3390/s20226577

**Published:** 2020-11-18

**Authors:** Haowei Dong, Xingshuang An, Yaodong Xiang, Fukai Guan, Qi Zhang, Qingqing Yang, Xia Sun, Yemin Guo

**Affiliations:** 1 School of Agricultural Engineering and Food Science, Shandong University of Technology, No. 266 Xincun West Road, Zibo 255049, China; 18503170269@stumail.sdut.edu.cn (H.D.); xsan2020@ytu.edu.cn (X.A.); 19403010081@stumail.sdut.edu.cn (Y.X.); 20403020269@stumail.sdut.edu.cn (F.G.); yqqing@sdut.edu.cn (Q.Y.); sunxia2151@sdut.edu.cn (X.S.); 2 Shandong Provincial Engineering Research Center of Vegetable Safety and Quality Traceability, No. 266 Xincun West Road, Zibo 255049, China; 3 Zibo City Key Laboratory of Agricultural Product Safety Traceability, Zibo 255049, China; 4 College of Life Science, Yantai University, No. 30 Qingquan Road, Yantai 264005, China; 5 Oil Crops Research Institute of the Chinese Academy of Agricultural Sciences, No. 2 Xudong 2nd Road, Wuhan 430062, China; zhangqi01@caas.cn

**Keywords:** deoxynivalenol, secondary antibody labeling, time-resolved fluorescence immunochromatography, field detection method, paper-based sensor

## Abstract

Immunoassay has the advantages of high sensitivity, high specificity, and simple operation, and has been widely used in the detection of mycotoxins. For several years, time-resolved fluorescence immunochromatography (TRFIA) paper-based sensors have attracted much attention as a simple and low-cost field detection technology. However, a traditional TRFIA paper-based sensor is based on antibody labeling, which cannot easily meet the current detection requirements. A second antibody labeling method was used to amplify the fluorescence signal and improve the detection sensitivity. Polystyrene fluorescent microspheres were combined with sheep anti-mouse IgG to prepare fluorescent probes (Eu-IgGs). After the probe fully reacted with the antibody (Eu-IgGs-Abs) in the sample cell, it was deployed on the paper-based sensor using chromatography. Eu-IgGs-Abs that were not bound to the target were captured on the T-line, while those that were bound were captured on the C-line. The paper-based sensor reflected the corresponding fluorescence intensity change. Because a single molecule of the deoxynivalenol antibody could bind to multiple Eu-IgGs, this method could amplify the fluorescence signal intensity on the unit antibody and improve the detection sensitivity. The working standard curve of the sensor was established under the optimum working conditions. It showed the lower limit of detection and higher recovery rate when it was applied to actual samples and compared with other methods. This sensor has the advantages of high sensitivity, good accuracy, and good specificity, saving the amount of antibody consumed and being suitable for rapid field detection of deoxynivalenol.

## 1. Introduction

Mycotoxins, which are widely found in various plant-derived foods, are harmful substances produced by the metabolism of fungi such as Aspergillus and Fusarium [1]. Mycotoxins contaminate agricultural products including grain, oil, vegetables, fruits, and nuts [2]. About 31 million tons of grain and oil are lost each year due to mycotoxins in China, and the direct economic loss is as high as 68–85 billion yuan [3]. Mycotoxins exceeding the standard contained in grain and oil products accumulate in the body after being ingested by humans and animals, causing carcinogenic changes and seriously endangering their health. At present, more than 300 mycotoxins have been identified, and deoxynivalenol as a common mycotoxin has attracted wide attention. Deoxynivalenol (DON), also known as vomiting toxin, is a derivative of fusobacterium graminea [4], mainly found in corn, wheat, and other food crops [5]. People and animals that consume excessive amounts of DON can experience nausea, diarrhea, vomiting, headache, fever, and other symptoms, hence the name “vomiting toxin” [6]. DON is highly cytotoxic and interferes with immune system development by inhibiting DNA and protein synthesis [7]. In view of the high harmfulness and widespread existence of DON, various countries have formulated corresponding limit standards.

There are many methods for the detection of DON. Instrumental analysis methods include high-performance liquid chromatography (HPLC) [8], Liquid chromatography-mass spectrometry/mass spectrometry (LC-MS/MS) [4], ultra-performance liquid chromatography (UPLC) [9], gas chromatography with mass spectrometry (GC-MS) [10], and high-performance liquid capillary electrophoresis (HPCE) [11]. HPLC is currently the most commonly used confirmative detection method for mycotoxins [12]. This method has the characteristics of high detection sensitivity, reliable results, and good specificity, and has been widely used in the detection of aflatoxin (AFT), DON, etc. However, most of the traditional instrumental analysis methods have the defects of high instrument price and complex pre-treatment process, and require specialized operators; thus, it is difficult to perform rapid field detection.

Developed in the 1950s, immunoassay is a qualitative and quantitative method for the detection of compounds, enzymes, and proteins, based on the specific reactions between antigens and antibodies. Immunoassay takes the antibody as the core recognition element, which can specifically bind to the corresponding target object. It has the advantages of high sensitivity, strong specificity, and simple operation [13,14], and has been widely used in the detection of DON. In recent years, time-resolved fluorescence immunochromatography (TRFIA) has attracted extensive attention among researchers due to advantages such as simple operation, fast detection, and low cost [15]. Using lanthanide elements europium (Eu), titanium (Ti), samarium (Sm), and other labeling materials, it replaces fluorescent dyes, enzymes, nano-gold, and other traditional labeling materials to label antibodies and other biological materials, and then carries out an immune reaction in the cellulose nitrate (NC) film region. Eu can emit orange fluorescence under ultraviolet lamp irradiation. “Time-resolved” refers to the detection and quantitative analysis of the signal strength of the object to be tested through wavelength resolution and time delay detection techniques. The most widely used TRFIA employs Eu, which has a long Stokes shift, a long fluorescence life, a narrow emission peak [16], and can avoid the interference of excitation light [17] after a certain chelation reaction. Zhang et al. [18] established aflatoxin TRFIA in agricultural products, and the detection range in peanut, rice, and vegetable oil was 0.8–25, 0.8–15, and 0.8–30 g/kg, respectively. The detection limit was 0.3 g/kg. This method showed good accuracy and precision, with the error of HPLC detection results being less than 10%, and good application prospects. Xiao et al. [19] created a rapid quantitative time-resolved fluorescence detection technology for DON, with a detection limit of 25 g/kg and a quantitative limit of 82 g/kg. The linear range of detection was 100–5000 g/kg, and the actual standard recovery of the sample was 83.51–113.84%. The technique showed good specificity, high sensitivity, good reproducibility, and simple operation, making it suitable for rapid quantitative detection of vomitoxin in grain feed. Wang Wenjun et al. [20] evaluated the system applicability of DON TRFIA, and the detection limit of this method was 154 g/kg, and the quantitative limit was 414 g/kg. There was no significant difference between the test results and LC-MS/MS, which confirmed the good stability and repeatability of the system.

However, with the improvement of people’s living standards and the constant adjustment of the limit standards of DON, traditional TRFIA based on the monoclonal antibody labeling method cannot easily meet the presentdetection requirements. In traditional methods, in order to improve the sensitivity of detection, the method of reducing the amount of antibody labeling is normally used. However, too low antibody labeling would reduce the coupling rate of fluorescent materials, leading to excessive background color of cellulose nitrate film during detection, which is prone to false negatives. Moreover, the conjugation of antibodies to fluorescent materials would reduce their ability to recognize antigens, which makes it difficult to meet our requirements. 

Therefore, in order to further improve the sensitivity of detection, a TRFIA paper-based sensor based on the secondary antibody labeling method was established. A sheep anti-mouse secondary antibody was coupled with a polystyrene fluorescent microsphere as the fluorescent probe, which was combined with the DON antibody, and the DON antibody was indirectly labeled on the fluorescent microsphere. In the traditional immune analysis strategy of targeting the antibody coupled with a microsphere, the microsphere can be coupled with multiple DON antibodies. During the coupling process, antibodies will lose some activity due to factors such as reagent and ultrasound, thus reducing their sensitivity. Conversely, in the secondary antibody labeling method, the activity and quantity of the secondary antibody are reduced when a secondary antibody is used instead of the DON antibody to conjugate to the microspheres; thus, the DON antibody is indirectly coupled to the microsphere with less quantity and higher activity. Compared with the traditional immune analysis, a small amount of DON antibody can show a strong fluorescence signal, thus improving the detection sensitivity. On the other hand, research shows that a single target antibody can be coupled with multiple secondary antibodies that are labeled on microspheres, resulting in the fluorescence signal intensity of the unit of antibody in the system being much higher than that in traditional methods [21,22]. Thus, the second antibody labeling method has the advantage of saving antibody consumption and avoiding the decrease in the ability of monoclonal antibodies to recognize antigens during the direct labeling process. Majdinasab et al. [21] successfully applied the secondary antibody labeling method in the detection of ochratoxin by TRFIA. Compared with the traditional antibody labeling method, the limit of detection (LOD) was 0.4 pg/mL, which increased by 100 times. Li [22] applied the secondary antibody labeling method for the detection of melamine and aflatoxin M1 in milk. It can be seen that the TRFIA based on the secondary antibody labeling method could improve the detection sensitivity compared with the traditional single-antibody method.

In this work, a TRFIA paper-based sensor based on the secondary antibody labeling method was established with DON as the target. The fluorescent probes (Eu-IgGs) were prepared by coupling sheep anti-mouse IgG with polystyrene fluorescent microspheres, and the antibody was connected to the Eu-IgGs indirectly. As the chromatography proceeded, the resulting compounds were spread out onto the paper sensor. Eu-IgGs-Abs that are not bound to the target are captured on the T-line, while those that are bound are captured on the C-line. The paper-based sensor reflected the corresponding fluorescence intensity change. With DON antibody as the core recognition element, the TRFIA for DON based on the secondary antibody labeling method was established. We explored the sensitivity, stability, accuracy, specificity, and other properties of this method, applied it to DON detection in corn and feed samples, and compared the findings with the analysis results of LC-MS/MS.

## 2. Experiment

### 2.1. Reagent

The sheep anti-mouse IgG and the rabbit anti-sheep IgG were obtained from Wuhan Baofu Biological Engineering Co., Ltd. (Wuhan, China). and Beijing Biodragon Immunotechnologies Co., Ltd. (Beijing, China), respectively. Europium oxide latex microspheres (size: 200 nm; 1%, m/v), sucrose, and Tween-20 were purchased from Sinopharm Group Chemical Reagent Co., Ltd. (Beijing, China). The reagents Na_2_HPO_4_·12H_2_O, NaH_2_PO_4_·12H_2_O, NaCl, KCl, and other chemicals were all analytically pure. Polyvinylpyrrolidone (PVPK-30) was purchased from Shanghai Youyou Biotechnology Co., Ltd. (Shanghai, China). Bovine serum albumin (BSA), 1-(3-dimethylaminopropyl)-3-ethylcarbonimide hydrochloride (EDC), DON-BSA, standards of mycotoxin (DON, AFM1, DON, T-2, zearalenone (ZEN), and FB1) were purchased from Sigma-Aldrich (St. Louis, Missouri, MO, USA). Cellulose nitrate film (NC film) and absorbent pad (CFSP 223000) were purchased from Millipore Corporation (Burlington, MA, USA). The DON antibody was obtained from our own laboratory. The glass cellulose film was produced by Shanghai Jining Biotechnology Co., Ltd. (Shanghai, China).

### 2.2. Instrument

The XYZ 3050 point-membrane apparatus and the CM 4000 strip cutters used to make the paper-based sensors were manufactured by Biodot (Irvine, CA, USA). Paper-based sensors were dried in a drying oven made by Yancheng Oulek Electronic Equipment Co., Ltd. (Shanghai, China). The CF 16RX high-speed refrigerated centrifuge used to obtain the required ingredients from the solution was made by Hitachi (Tokyo, Japan). A time-resolved fluorescence speedometer for rapid quantitative detection of substances was produced from Shanghai Youyou Biotechnology Co., Ltd. (Shanghai, China). The LC-MS/MS used for quantitative analysis of samples was manufactured by Shimadzu (Kyoto, Japan). All the aqueous solutions used in the experiment were prepared by LS MK2 Pall ultrapure water system (18.2 MΩ·cm, New York, NY, USA). The fluorescent probes were characterized by a spectrophotometer produced by Seymour Fisher Technology Co., Ltd. (Iowa, USA).

### 2.3. Preparation of Fluorescent Probes (Eu-IgGs)

100 μL polystyrene fluorescent microspheres were added to 400 μL boric acid buffer (0.2 M, pH 8.18). After vortex mixing, the polystyrene fluorescent microspheres were evenly dispersed in the boric acid buffer by ultrasonic stirring for 10 min. A certain amount of EDC solution (15 mg/mL) was added and eddy-mixed for 15 min to activate the carboxyl group on the polystyrene fluorescent microsphere particles for the convenience of antibody coupling. The mixed solution was centrifuged at high speed (13,300× *g*, 10 °C, 10 min), the supernatant was discarded, and the excess EDC was removed. The precipitation was remixed with 500 μL boric acid buffer, and the polystyrene fluorescent microspheres were fully mixed with ultrasonic for 10 min. An appropriate amount of sheep anti-mouse IgG was added to the redissolved solution, which was oscillated at low temperature for 12 h. The supernatant was removed by centrifugation, and 1 mL boric acid buffer containing BSA solution (0.5%, m/v) was added to seal the non-specific binding sites on the surface of the polystyrene fluorescent microspheres. The solution shaker reaction took place at 20 °C for 3 h. The prepared probes (Eu-IgGs) were stored at 4 °C.

### 2.4. Preparation of Paper-Based Sensors

**Figure 1:**
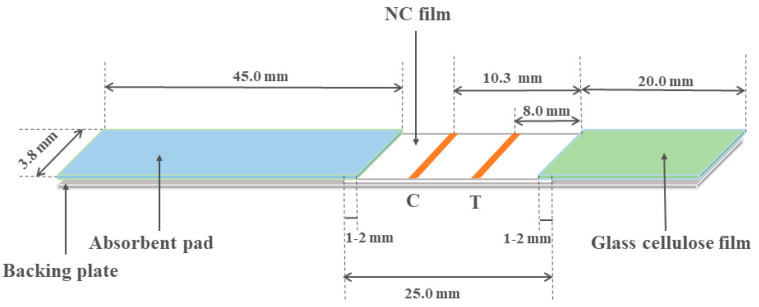
Time-resolved fluorescence immunochromatography (TRFIA)paper-based sensor assembly structure diagram.

The glass cellulose film sealing solution was prepared: 2.9 g NaH_2_PO_4_·12H_2_O, 0.3 g NaH_2_PO_4_·12H_2_O, 1.0 g PVPK-30, 0.5 g BSA, 1.0 g Tween-20, and 0.25 g ethylenediaminetetraacetic acid (EDTA) were weighed and dissolved in 100.0 mL ultra-pure water. The TRFIA paper-based sensor was mainly composed of four parts: backing plate, cellulose nitrate film (NC film), glass cellulose film, and absorbent pad. The XYZ3050 strip sprayer was cleaned with methanol and water, respectively, to ensure smooth water flow before marking. At a spraying rate of 0.7 μL/mL, line C (rabbit anti-sheep IgG) and line T (DON-BSA) were sprayed from one end of the absorbent pad to the other end of the glass cellulose film. After the marking was completed, the bottom plate was dried at 37 °C for 2 h. The glass cellulose film was soaked in the glass cellulose film sealing solution for 15 min and then removed and dried in the oven at 37 °C for 2 h. The absorbent pad was combined with the dried bottom plate and glass cellulose film. As shown in Figure 1, the glass cellulose film, NC film, and absorbent pad were successively pasted on the backing plate, with each component overlapping 1 mm. Placed at 4 °C for 20 min, after the bonding was complete, the strips were cut into paper-based sensors with a cutting machine according to 3.8 mm/strip. The prepared TRFIA paper-based sensor was sealed and stored at 4 °C.

### 2.5. Working Method of the TRFIA Paper-Based Sensor

Sucrose can increase hydrophobicity and chromatography speed. BSA can block and reduce non-specific binding sites. Tween-20 can improve the stability of reaction and the specific recognition ability of immunoreagents. PVPK-30 has good dispersibility and can eliminate the background color of NC film. According to the literature [23], sucrose, BSA, Tween-20, and PVPK-30 were successively added to ultra-pure water with volume ratios of 1%, 0.5%, 2%, and 1% to obtain the reaction sustained release solution. Time-resolved fluorescence detection was performed by immunochromatographic strips and time-resolved fluorescence speedometer. An appropriate amount of the reaction sustained release solution, Eu-IgGs, antibodies, and sample extracts were added to the sample cup to make the total volume of the reaction 160 μL. After being incubated at 37 °C for 10 min in a constant temperature incubator, the prepared paper sensor was inserted into the sample cup. After incubation, the Eu-IgG was deposited onto the NC film along the glass cellulose film, and an indirect competitive immune response occurred in the reaction area.

**Figure 2:**
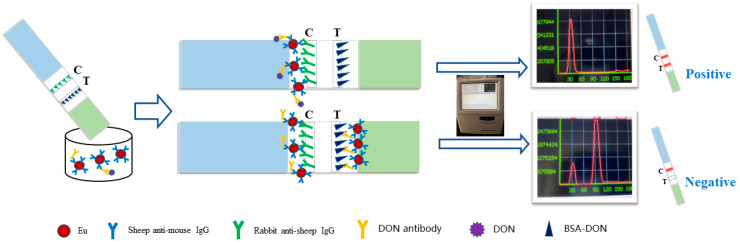
Working principle of TRFIA paper-based sensor based on secondary antibody labeling.

When there was no detection target in the sample, the DON antibody carried Eu-IgGs by capillarityto the line T position and bound to the DON-BSA while the remaining Eu-IgG bounded to the rabbit anti-sheep IgG located on line C. At this time, the fluorescence intensity on line T was the highest, and line C was the lowest. The ratio of the fluorescence signal value of line T to the fluorescence signal value of the C line (T/C) was the highest, and the test result was negative. When the sample contained a small amount of the detection target, the antibody reacted with the target first. The unbound antibody carried Eu-IgGs and bound to DON-BSA on the T line by chromatography. When the antibody combined with the target object carried Eu-IgGs by chromatography to line C, the fluorescence signal of the T line was weakened, the fluorescence signal of line C was strengthened, and the T/C value was correspondingly reduced. When the sample contained too much detection target, the antibody was consumed by the target, unable to carry Eu-IgGs combined with chromatography to line T, and Eu-IgGs gathered on line C. At this time, line T signal was the weakest, the C line signal was the strongest, the T/C value was the smallest, and the test result was positive. The concentration of the target in the reaction solution was inversely proportional to the T/C value of the fluorescence band. The reaction is shown in Figure 2.

### 2.6. Sample Pretreatment Method

Samples of 5g of corn and feed, respectively, were ground after crushing. 20 mL methanol solution (70%, v/v) was added into the matrix and extracted for 30 min with 0.45 μm organic phase membrane filter. We took 1 mL and added it to 4 mL of the reaction slow-release fluid filtrate (diluted 5 times). The filter liquor was added to the sample diluent and be used for TRFIA paper-based sensors for testing. 

## 3. Results and Discussion

### 3.1. Characterization of Eu-IgGs

**Figure 3:**
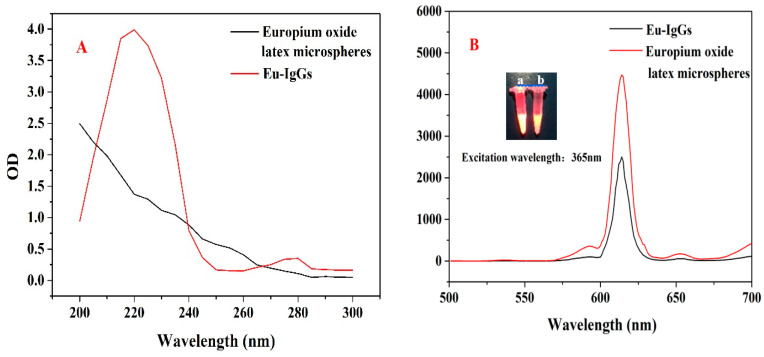
(**A**) Uv-vis absorption spectra of europium oxide latex microspheres before and after sheep anti-mouse IgG conjugation; (**B**) Fluorescence spectra of europium oxide latex microspheres before and after conjugation to sheep anti-mouse IgG; the illustration is a fluorescence picture of the europium oxide latex microspheres before (a) and after (b) the conjugation to sheep anti-mouse IgG.

In order to prove the successful binding of europium oxide latex microspheres with sheep anti-mouse IgG, we scanned the blank europium oxide latex microspheres and the conjugated europium oxide latex microspheres with sheep anti-mice using UV-vis spectroscopy (Figure 3A). We observed that there was no wave peak in the blank europium oxide latex microspheres, but there were obvious wave peaks in the sheep anti-mouse IgG latex microspheres around 220 nm and 260 nm. It was proved that europium oxide latex microspheres were successfully combined with sheep anti-mouse IgG. We used fluorescence spectrometry to scan the europium oxide latex microspheres before and after the sheep anti-mouse IgG conjugation, and the maximum emission peak was observed at 614 nm (Figure 3B). The fluorescence signal was partially weakened after the sheep anti-mouse IgG coupling. The reason was that europium oxide latex microspheres formed a complex with sheep anti-mouse IgG, resulting in fluorescence quenching.

### 3.2. Condition Optimization of TRFIA Paper-Based Sensor

**Figure 4:**
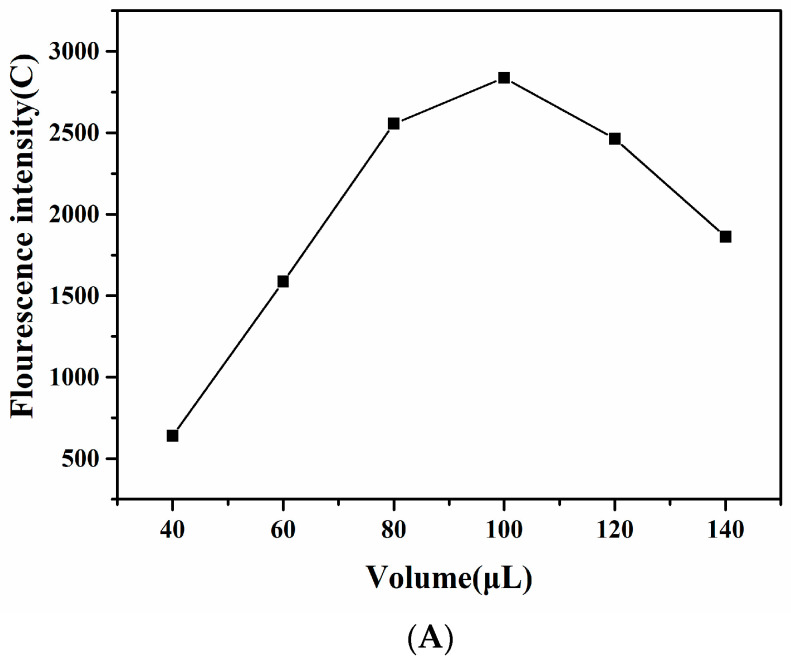

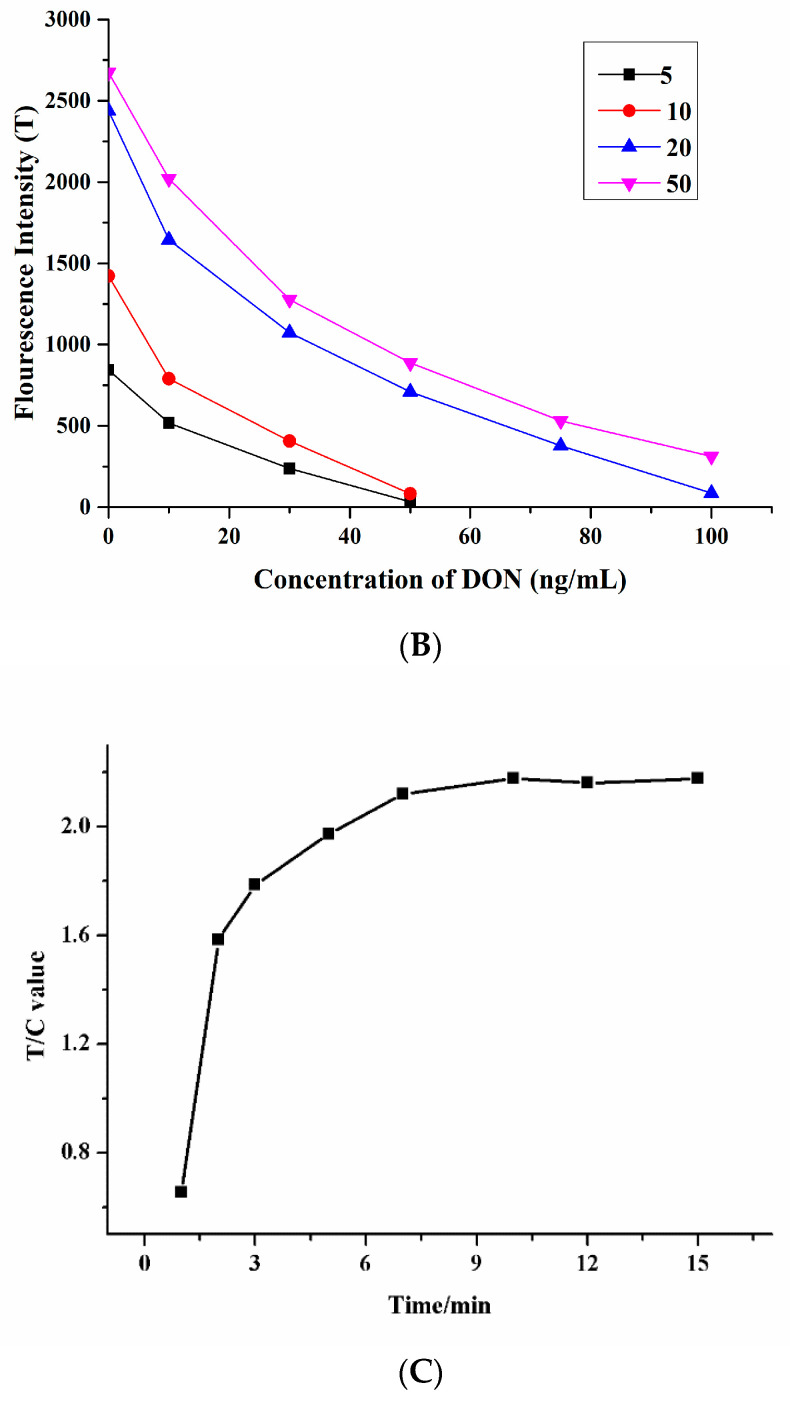
(**A**) Optimization of coupling amount of sheep anti-mouse IgG; (**B**) Different Deoxynivalenol antibody dosages and fluorescence intensity of line T; (**C**) Optimization of reaction time.

The coupling amount of sheep anti-mouse IgG could indirectly affect the sensitivity and detection range of the paper-based sensor. As shown in Figure 4A, the fluorescence intensity of line C increased with the increase in the amount of anti-mouse IgG (1 mg/mL) in the range of 40–100 μL. However, the fluorescence intensity of line C decreased when the dosage of secondary antibody exceeded 100 μL. This indicated that the coupling amount of sheep anti-mouse IgG had reached saturation. When the amount of anti-mouse IgG of sheep was too much, the amount of Eu-IgGs carried by the unit of antibody would decrease. In the process of paper-based sensor operation, the higher the concentration of Eu-IgGs was, the stronger the fluorescence value carried by the unit of antibody became, and the higher the fluorescence value of line C became, which could reduce the loss of DON antibody. Therefore, we choose 100 μL as the optimal of anti-mouse IgG conjugate dose, which made line C steady.

The amount of DON antibody directly determined the sensitivity and detection range of the method. In order to ensure detection sensitivity, the amount of DON antibody needed to be controlled. We optimized the system with different amounts of DON antibodies. In negative samples, when the concentration of DON antibody was higher, more Eu-IgGs would be bound, and the fluorescence intensity of line T was higher. In positive samples, DON would consume the corresponding DON antibody, leading to a decrease in the fluorescence intensity of line T. In other words, reducing the amount of DON antibody can improve sensitivity, but it would lead to a narrower detection range and false positive or false negative cases. An excess of DON antibodies, on the other hand, decreased sensitivity. When the concentration of DON was high, the antibody fully bound to the target. At this point, line C was saturated and line T was unable to capture the complex. As known, after line C reaches saturation, the addition of too much sheep anti mouse IgG was not of any positive significance. Thus, this concerns the “optimum concentration,” which is the concentration of IgG that is just enough to saturate line C. According to the experiment, as shown in Figure 4B, when the DON concentration was 100 ng/mL, the fluorescence signal of line T just disappeared when antibody (20 ng/mL) was added. Therefore, 20 ng/mL was the optimal amount of DON antibody. As shown in Figure 4C, the T/C value gradually increased as the reaction progressed. As can be seen from the figure, when the reaction time was 10 min, the T/C value reached the maximum and did not change within a short time, indicating the reaction reached equilibrium. Therefore, the response time of the paper-based sensor should be at least 10 min.

### 3.3. Detection of DON

**Figure 5:**
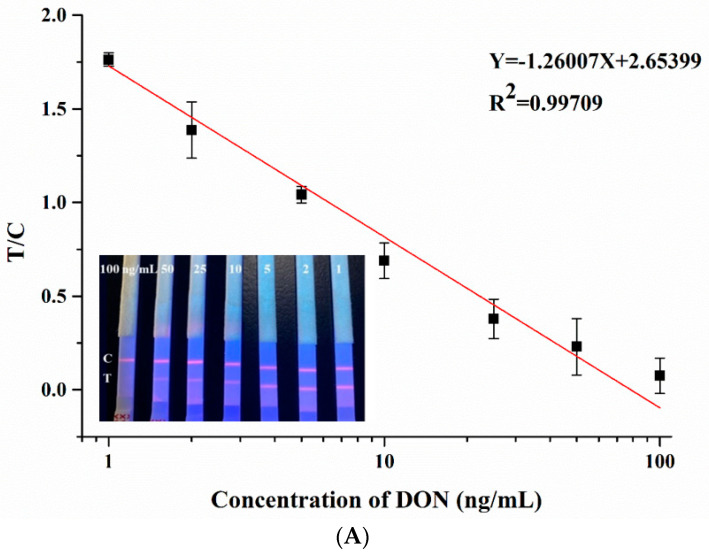

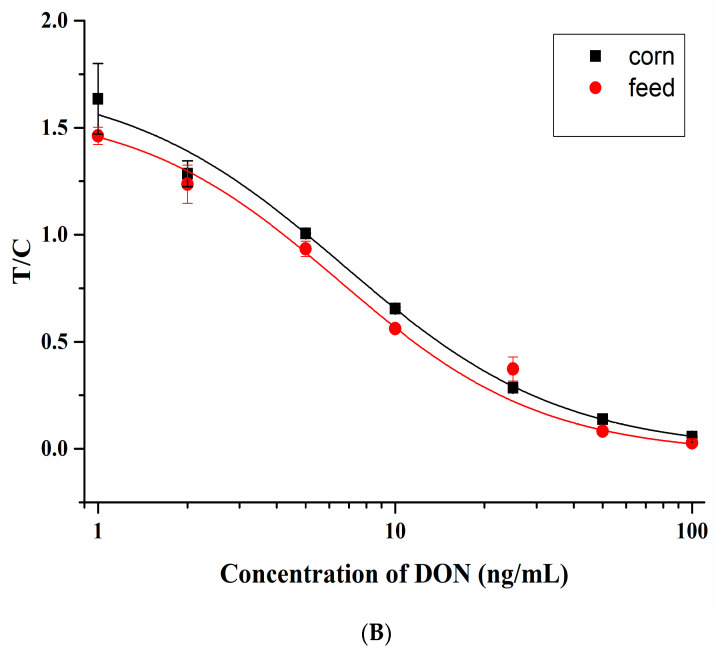
(**A**) Standard curve of quantitative detection in 70% methanol (Illustration: physical image of paper-based sensor with a series of pesticide concentrations added); (**B**) Standard curve of quantitative detection in corn and feed samples.

We prepared three different blank sample solutions: methanol solution (70%, *v*/*v*), corn matrix, and feed matrix. The solution of the prepared blank sample tested negative by LC-MS/MS. The working linear equation of the paper-based sensor was established by quantitatively adding the DON standard to the blank sample solution. Each labeled concentration was repeatedly measured five times. The fluorescence band reading was performed with a time-resolved fluorescence detector. To reduce the experimental error, we choose T/C as the independent variable. With a certain amount of Eu-IgGs added, such a strategy can detect the change of line C and line T at the same time, so as to reflect the change in the number of targets more accurately. Under the best experimental conditions, the relationship between the DON concentration (X) and the T/C value (Y) was studied. After 20 measurements of negative samples, the mean value (B0) and standard deviation (SD) were obtained. The LOD could be obtained by substituting into the equation (LOD = B0 - 3*SD). As shown in Figure 5A, the standard working equation of DON in methanol solution (70%, *v*/*v*) was Y = −1.26007X + 2.65399, and R^2^ could be 0.99709. The LOD of DON in methanol solution (70%, *v*/*v*) was 0.121 ng/mL, with a good linear range (1–100 ng/mL). The LOD was 0.206 ng/mL in the maize matrix, and 0.216 ng/mL in the feed matrix (Figure 5B). As seen from Figure 5, with the increase in DON concentration, the fluorescence signal value of line T on the strip gradually decreased.

The reason was that the concentration of DON in the sample increased, the competitive inhibition effect of DON-BSA on line T was enhanced, and the consumption of DON antibody increased. This resulted in a decrease in the amount of Eu-IgGs fixed on line T, which in turn reduced the brightness of line T. When the concentration of the target exceeded the detection limit, the T line completely disappeared. This indicated that the DON antibody in the sample solution was completely consumed, and no Eu-IgG was fixed on line T.

Compared with instrumental analysis, immunochromatography had the advantages of simple operation, low cost, and suitability for field detection. In this work, the TRFIA paper-based sensor based on the secondary antibody labeling showed lower sensitivity compared with other immunochromatography methods (Table 1).

### 3.4. Performance Test of Paper-Based Sensor

**Figure 6:**
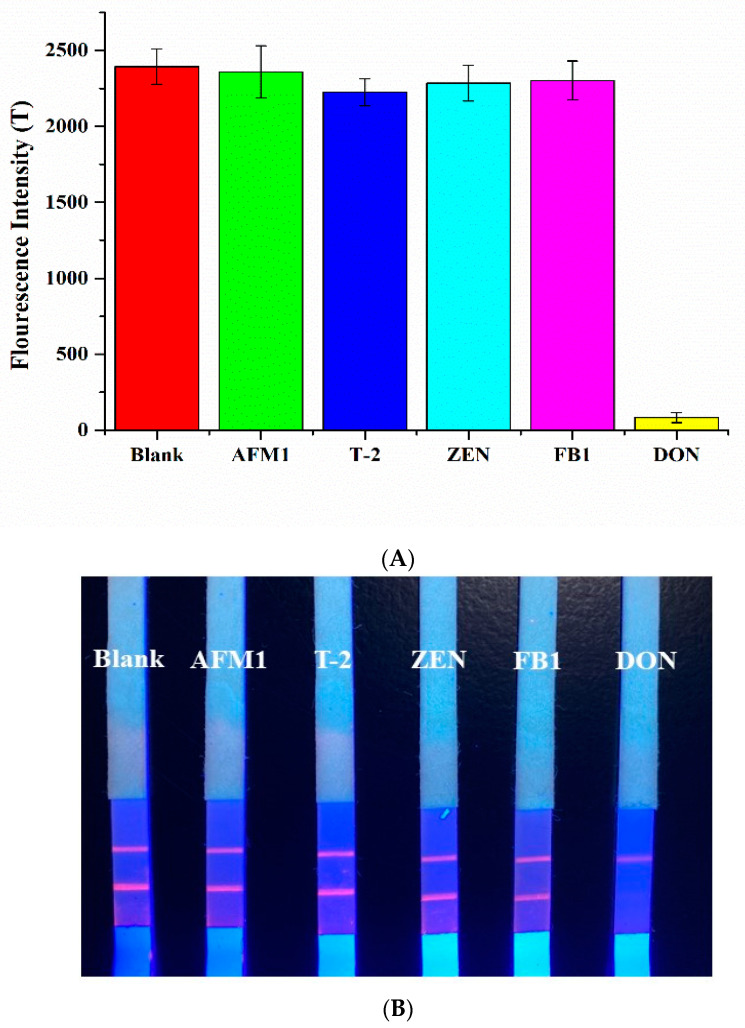
Paper-based sensor specificity evaluation (**A**) and physical images (**B**).

Specificity is one of the important properties of sensors. As shown in Figure 6, blank samples, AFM1, DON, T-2, ZEN, and FB1 were used to conduct specificity tests of paper-based sensors. The fluorescence intensity of line T was read with a time-resolved fluorescence detector. The results showed that the TRFIA paper-based sensor based on secondary antibody labeling had good specificity in the detection of DON. As could be seen in Figure 5B, when the non-target toxin was added, the fluorescence intensity of line T hardly changed compared with the blank sample. When the target toxin was added, the line T strip disappeared.

High, medium, and low concentrations of the DON standard were added to corn and feed samples to evaluate sensor accuracy and precision. The accuracy of the method was examined by calculating the recovery rate, and the precision of the method was examined by the coefficient of variation. As shown in Table 2, the recoveries of maize samples ranged from 88.07% to 121.22%, and the relative standard deviation was lower than 10.49%. In the feed samples, the recovery rate was 94.8–107.49%, and the relative standard deviation was lower than 8.74%. In this study, the TRFIA paper-based sensor based on secondary antibody labeling for DON had a high recovery rate and good precision, which could meet the actual detection requirements.

### 3.5. Detection of DON in Samples

The paper-based sensor was applied to the detection of DON in corn and feed samples, and compared with the results of LC-MS/MS. As shown in Table 3, the recovery rate was 80.24–117.4%, with good recovery results.

## 4. Conclusions

In this work, polystyrene fluorescent microspheres were directly coupled with sheep anti-mouse IgG, and then indirectly coupled with DON antibody to prepare fluorescent probes. The working conditions, such as antibody coupling amount, DON antibody dosage, and reaction time, were optimized. Under optimal working conditions, the TRFIA paper-based sensor based on secondary antibody labeling was constructed. The detection range of the paper-based sensor was 1–100 ng/mL, and LOD could reach 0.121 ng/mL. Moreover, its LOD in the maize matrix and feed matrix was 0.206 ng/mL and 0.216 ng/mL, respectively. The recoveries remained within the range of 88.07–121.22%, and the relative standard deviation was lower than 10.49%. Compared with the LC-MS/MS method, the recovery rate of this work could be maintained at 80.24–117.4% when testing actual samples. All of the above indicated that the developed sensor has good sensitivity, specificity, precision, and accuracy. Compared with the instrumental analysis method, this sensor has the advantages of simple operation, short detection time, and low detection cost. Compared with other rapid detection methods, it has the advantages of high sensitivity and saving on the amount of monoclonal antibody. This paper-based sensor could be applied to the rapid field detection of large quantities of samples. According to this work, in the future, we will consider fixing multiple lines T (i.e., antigens of various mycotoxins) on a paper-based sensor with the support of optimizing more operating parameters and corresponding data reading devices, so that it is possible to detect a variety of mycotoxins simultaneously.

## Figures and Tables

**Table 1:** Comparison of the present study with existing detection technologies.

Detection Technologies	Limit of Detection (LOD) (ng/mL)	References
Lateral-flow immunochromatographic assay strip	1.97–46.8	[24]
Simultaneous surface plasmon resonance	3.26	[25]
Colloidal gold immunochromatographic assay	25	[26]
Direct competitive fluorescent-labeled immunosorbent assay	5.6	[27]
Flower-like gold nanoparticles-based immunochromatographic test strip	5	[28]
Immunochromatographic test card (DON-GICT)	40	[29]
HPLC-UV	4.4	[30]
Immunochromatographic assay using secondary antibody-europium nanoparticle conjugates	0.121	This work

**Table 2:** Spike recovery experiment of corn and feed samples.

Samples	Spiked (ng/mL)	Detected (ng/mL)	Recovery (%)	Relative Standard Deviation (RSD) (%)
Corn	10	8.81	88.07	8.23
	50	60.61	121.22	10.49
	80	85.26	106.58	6.98
Feed	10	10.47	104.67	8.74
	50	53.74	107.49	8.28
	80	75.84	94.8	3.09

**Table 3:** Detection of DON in samples.

Samples	LC-MS/MS (ng/mL)	Paper-Based Sensors (ng/mL)	Recovery (%)
Corn 1	65.96	58.16	88.17
Corn 2	33.19	26.63	80.24
Corn 3	47.03	55.21	117.40
Feed 1	26.97	31.02	115.02
Feed 2	80.21	72.87	90.85
Feed 3	55.76	52.35	93.88
